# Factors associated with children’s health facility visits for primaquine treatment in rural Papua New Guinea

**DOI:** 10.1186/s12936-019-2675-4

**Published:** 2019-02-20

**Authors:** Akiko Yoshii, Makoto Sekihara, Takuro Furusawa, Francis Hombhanje, Takahiro Tsukahara

**Affiliations:** 1 TWMU Career Development Center for Medical Professionals, Tokyo Women’s Medical University, 8-1 Kawada-cho, Shinjuku-ku, Tokyo, 162-8666 Japan; 2 Department of International Affairs and Tropical Medicine, Tokyo Women’s Medical University, 8-1 Kawada-cho, Shinjuku-ku, Tokyo, 162-8666 Japan; 3 Department of Tropical Medicine and Parasitology, Juntendo University School of Medicine, 6th Floor, Bldg. No.9, 2-1-1, Hongo, Bunkyo-ku, Tokyo, 113-8421 Japan; 4 Department of Ecology and Environment, Graduate School of Asian and African Area Studies, Kyoto University, Room#AA431, Research Bldg. No.2, Yoshida-Honmachi, Sakyo-ku, Kyoto, 606-8501 Japan; 5 Centre for Health Research and Diagnostics, Divine Word University-Rabaul Campus, Kokopo, Papua New Guinea; 6 School of Economics, Hosei University Graduate School, 2-15-2 Ichigaya Tamachi, Shinjuku-ku, Tokyo, 162-0843 Japan

**Keywords:** Antimalarials, Therapeutic uses, Child, Vivax malaria, Continuity of patient care, Health service accessibility, Patient compliance, Patient acceptance of health care, Appointments and schedules

## Abstract

**Background:**

To control and eventually eliminate vivax malaria, radical treatment with primaquine (PQ) is essential after completion of blood-stage treatment. Although in many malaria-endemic countries, village health volunteers (VHVs) are engaged in diagnostic treatment of malaria in remote communities, they principally provide blood-stage treatment. In such a situation, access to PQ following blood-stage treatment can be a barrier to complete treatment. However, studies on access to PQ treatment have been scarce and limited in health facility-based settings. This study aimed to identify factors associated with access to PQ treatment in rural Papua New Guinea (PNG) from the community case management perspective.

**Methods:**

A community-based, cross-sectional survey was conducted to collect sociodemographic information on children under 15 years of age, their households, and their caretakers in East Sepik Province, PNG. Data collection lasted from February to March, 2015. Information on the diagnoses of potential non-falciparum malaria and prescription of PQ in preceding year (January to December 2014) were obtained from child health-record books. Then, multilevel logistic regression model was used to determine the factors associated with formal health facility visits for PQ treatment among children with potential non-falciparum malaria.

**Results:**

Of 420 episodes diagnosed as potential non-falciparum malaria, 46 (11%) were immediately given PQ. The rest were instructed to visit formal health facilities (HFs) for PQ, and the patients obtained PQ during the second visit to HFs was 44%. Consequently, the overall proportion of PQ prescription was 50%. Logistic regression analysis suggested that among the patients who were instructed to visit HFs for PQ treatment, the initial visit to VHV and higher transportation costs to HF were inversely associated with PQ prescription during the second visit to an HF.

**Conclusions:**

Few children received PQ treatment during the second visit to HFs following diagnosis of potential non-falciparum malaria. These findings suggest a need to establish a policy to reduce structural and economic barriers and improve rural inhabitant access to PQ treatment.

**Electronic supplementary material:**

The online version of this article (10.1186/s12936-019-2675-4) contains supplementary material, which is available to authorized users.

## Background

With the global effort to control malaria, the relative proportion of *Plasmodium vivax* tends to increase in regions where *P.* *vivax* and *Plasmodium falciparum* are co-endemic [1]. In countries outside Africa, vivax malaria accounts for 36% of total malaria cases [2]. Unlike *P.* *falciparum*, *P.* *vivax* can remain dormant in the liver as hypnozoites, reemerging to cause recurrent symptoms and new transmissions, which comprises a major obstacle to control this disease. Primaquine (PQ) is the most standard and approved efficacious drug used against hypnozoites.

In Asia–Pacific countries, *P.* *vivax* is dominant and the region accounts for over 80% of *P. vivax* cases other than Africa [3]. In 2009, the Asia–Pacific Elimination Network (APMEN) was established especially focusing on controlling *P. vivax* with 18 affiliate countries [4]. Although, in all member states, PQ has been introduced as a component of the standard treatment of vivax malaria [2], information on the effectiveness of PQ treatment in routine clinical settings is sparse. Previous studies have focused mainly on adherence to 14 days’ administration of PQ with varied adherence rate from 24 to 98% [5–8]. In addition, these studies showed that males, children, side effects, oral instruction for treatment, and improvement or worsening of symptoms tended to be related to nonadherence.

In many malaria-endemic countries, village health volunteers (VHVs) are often the first point of contact with medical services. Among APMEN countries, 11 countries have deployed VHVs for malaria diagnosis and treatment in remote communities [9–19]. However, the VHVs have generally engaged in blood-stage treatment of vivax malaria, and have not been allowed to prescribe PQ because of safety concerns about severe acute haemolytic anaemia in glucose-6-phosphate dehydrogenase (G6PD)-deficient patients. Although the possibility of PQ delivery by VHVs was discussed in the international meeting for vivax elimination in 2016, the current guideline of the World Health Organization (WHO) does not endorse this option [4].

In Papua New Guinea (PNG) one of the APMEN countries, the different Plasmodium species co-exist, and *P. vivax* is a major contributor to high morbidity rates for malaria, especially in young children [20, 21]. The incidence of *P.* *vivax* malaria is seasonal and reaches a peak in the early rainy season, from October to November [21]. A new national malaria-treatment protocol was officially announced in 2009, and parasite-based diagnoses using microscopy or rapid diagnostic testing became required instead of symptom-based diagnoses. In our previous study in PNG, of the febrile children who sought treatment outside the home, 60% had first consulted VHVs [15]. However, PQ has been provided only in formal health facilities where health providers can clinically assess a patient for potential PQ-induced haemolysis, and children diagnosed with vivax malaria are instructed to visit HFs to obtain PQ after blood-stage treatment with artemether–lumefantrine (AL) in a community level.

In such a situation, access to PQ treatment during the second visit to an HF following blood-stage treatment can be a barrier to complete the treatment. However, previous studies on access to PQ treatment have been scarce, and limited in health facility-based settings [22–24]. Therefore, access to PQ treatment was investigated, including referrals from VHVs to HFs in an actual low-resource setting, as well as associated factors from the community case management perspective, to propose policy recommendations for controlling vivax malaria in the Asia and Pacific region.

## Methods

### Study site

The study was conducted in the Boiken Dagua Rural Local Level Government area of Wewak district, located on the northern coast of East Sepik Province, PNG. In East Sepik Province, *P.* *vivax* is the second-most prevalent parasite after *P.* *falciparum*; the overall mean prevalence of *P.* *vivax* was 35.7% [20]. In PNG, non-governmental organizations have promoted the deployment of VHVs since the early 2000s [25–27]. There were 23 villages in the study area, and one VHV was deployed in each village. Details of VHV framework have been previously described [28]. The term “health facility” (HF) was used to define a formal facility operated by health professionals approved to prescribe PQ. At the time of this study, five HFs (one health centre and four aid posts) were operated in the study area.

High-grade *P. vivax* chloroquine (CQ) resistance was reported in PNG in the 1990s [29, 30], therefore, combinations of CQ or amodiaquine with sulfadoxine-pyrimethamine (SP) for *P.* *vivax* were introduced in 2002. Because of the subsequent rapid increase in SP resistance, a drug efficacy trial comparing AL, dihydroartemisinin–piperaquine (DHA–PPQ), and CQ + SP was conducted between 2005 and 2007 [31]. This showed that the AL regimen was the most efficacious against *P.* *vivax,* followed by the DHA–PPQ, and CQ + SP regimens. For these reasons, AL became a first-line treatment for vivax malaria in the new national malaria-treatment protocol in 2009 [32].

Since then, PQ, which the former guideline recommended only for vivax malaria patients in low malaria-endemic regions [33], has become a standard treatment for all patients diagnosed with uncomplicated vivax and/or ovale malaria after completing 3 days of treatment with AL. If a patient’s G6PD deficiency status is unknown, health providers should advise the patient to stop PQ and return to the health facility if they pass dark-coloured urine, or they should clinically assess the patient for a minimum of 1 h following the administration of PQ [34]. Following WHO recommendations [35], a dose of 0.25 mg/kg daily for 14 days was adopted for radical cure of *P.* *vivax* in the PNG guideline. In the study area, a rapid diagnostic test (RDT; *CareStart™* Malaria HRP2/pLDH (pf/PAN) Combo, Access Bio Inc., USA) was widely used instead of microscopic diagnosis, and PQ treatment was recommended for patients diagnosed with “potential non-falciparum malaria”, i.e., “a *non*-*P. falciparum,*” “*P. falciparum,* or *P. falciparum* with another species.”

### Study population and data collection

A community-based, cross-sectional survey using a structured questionnaire was conducted during February and March 2015. The study population included children aged 0 to 14 years living in coastal lowlands of the catchment area of Dagua Health Centre. The area consisted of 23 villages with 1056 households and 2767 children. A structured questionnaire was used for collecting information, and it was written in English (one of the official languages of PNG). Coauthors (TT, MS and TF) were able to communicate in the local language (Tok Pisin: lingua franca and another official language of PNG) and they instructed field assistants on the intended meaning of each question and how to administer the questionnaire in Tok Pisin. Trained field assistants interviewed caregivers of the target children to collect sociodemographic information. Medical records of malaria diagnosis results and subsequent treatment regimens for study participants during the preceding year were also collected (January to December 2014). Individual outpatient records were not kept in the study area’s HFs. Instead, VHVs, as well as medical personnel of HFs are ordered to fill out medical information in a child health-record book, which each patient retained at home and was expected to bring to each VHV/HF visit. For that reason, trained field assistants visited each household and collected child health-record books; the relevant pages from these books were photocopied and then returned to the caregivers. Then, the necessary information for the study was encoded and was single-entered into a database table.

### Data analysis

Following local guidelines, episodes with potential non-falciparum malaria were included for the logistic regression analysis. The dependent variable was “access to PQ”, defined as the PQ prescription during the second visit to HFs following diagnosis of potential non-falciparum malaria. The child’s gender, child’s age, caregiver’s age, caregiver’s education level, caregiver’s marital status, total number of residents in the household, durable assets in the household, place of initial visit (an HF or a VHV), and transportation costs were identified as independent variables.

In this study, the caregiver’s education level was specified as the total years of schooling. Household size was defined as the total number of residents in the household. On the basis of 13 dummy asset variables (pig, chicken, clock, mobile phone, radio, generator, chain saw, solar panel, car, bicycle, computer, television, and refrigerator), an asset index, as a predictor of household economic status, was calculated using principal component analysis (PCA) [36, 37]. The asset index was scored on the basis of the first principal component. Thereafter, these scores were divided equally into three categories: upper, middle and lower.

Transportation cost was defined as the cost of return fare of transport vehicle from the nearest roadside to HFs. There was no charge to receive PQ, including related consultation and treatment costs, at HFs, so the direct cost to patients and their caregivers equaled only transportation cost.

All data were entered into a Microsoft Access 2013 database and were then analysed using Stata SE14 (Stata Corp., College Station, TX, USA). After the characteristics of the children, caregivers, and households surveyed were entered, a three-level multilevel random intercept logistic regression model was used to identify the association between PQ prescription during the second visit to an HF and those characteristics. In the analysis, potential non-falciparum malaria episodes (level 1) were nested in an individual (level 2) and individuals were nested in a village (level 3). Wilcoxon rank-sum tests were used to compare medians of continuous variables, and Chi-square tests were applied to compare proportions of categorical variables between the groups. The level of statistical significance was set at *p* < 0.05.

## Results

### Potential non-falciparum malaria episodes for multilevel logistic regression analysis

This study population included 2767 children under age 15 registered in the household questionnaire survey. Of these, 30% (835/2767) were excluded because of uncollected child health-record books: children had no book (16% [437/2767]); children had moved out of the community at the time of record-book collection (11% [312/2767]); caregivers declined to provide a child health-record book (3% [86/2767]). Descriptive statistics of the characteristics of children in the excluded group (n = 835) were compared with those in the remaining group (n = 1932). There were significant differences in the age of the patients, the age of the caregivers, the marital status of caregivers, household size, and wealth status between the two groups (Additional file 1: Table S1).

**Fig. 1:**
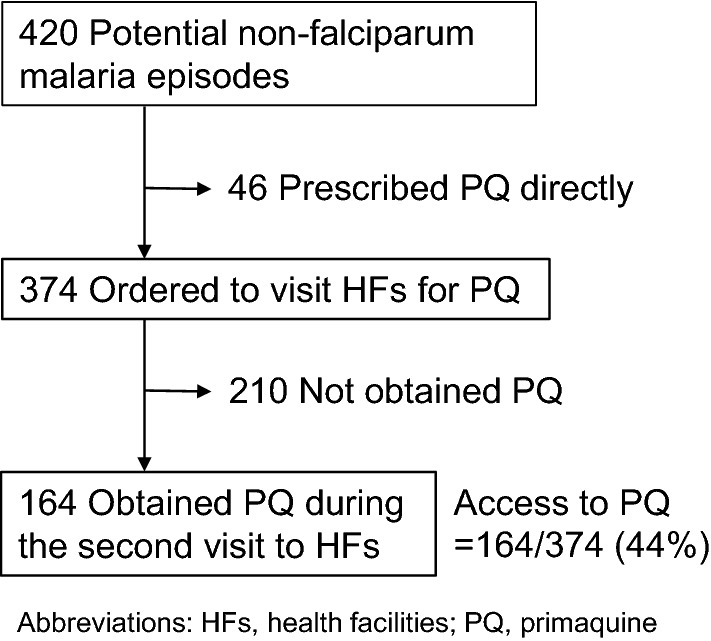
Access to PQ treatment. Access to PQ treatment was calculated as the ratio between the number of patients with potential non-falciparum malaria who received PQ during the second visit to HFs and the number directed to visit HFs

Among the remaining 1932 children, 684 were examined for malaria infection using RDT at least once during the study period. A total of 1059 RDTs were performed. Of these, 639 (60%) were excluded (77 had a *P.* *falciparum* single infection, 437 had negative RDT results, 125 had improper or unrecorded RDT or PQ prescription results). Among the remaining 420 episodes diagnosed as potential non-falciparum malaria, 50% (210/420) were prescribed PQ including 46 episodes (11%) which were directly prescribed PQ at their initial medical care. Finally, 374 potential non-falciparum malaria episodes were selected for the logistic regression analysis (Fig. 1).

### PQ prescription during the second visit to HF

Of 374 potential non-falciparum malaria episodes in which children were directed to revisit HFs for PQ treatment, 164/374 (44%) were prescribed PQ during the second visit to HFs (Fig. 1). The median between the first visit (when blood-stage treatment with AL commenced) and the second visit to HFs (when patients obtained PQ treatment) was 4 days (interquartile range [IQR] 3, 6) in 164 episodes. Among 210 episodes with no PQ prescription, four visited HFs within 14 days after diagnosis of potential non-falciparum malaria. Some children had multiple potential non-falciparum malaria episodes during the study period. On average, 1.2 potential non-falciparum malaria episodes per child (374 episodes in 309 children) were seen, with a maximum of three episodes in one child.

### Characteristics of children, their caregivers, and their households

Descriptive statistics of the characteristics of children included in the analysis are shown in Table 1. Of the 309 children who received at least one diagnosis of potential non-falciparum malaria, 54% were boys, and the median age was 7 years (IQR 4, 10). Most of caregivers were married (92%), and the median caregiver age was 34 years (IQR 28, 42). The median number of years of education was 6 (IQR 6, 8). The median household size was 6 (IQR 5, 7), with a maximum of 17 people in one household. The median transportation cost to HFs was 0 kina ([IQR 0, 4], Kina 1 = USD 0.37 in 2015).

**Table 1:** Sociodemographic characteristics of children with potential non-falciparum malaria who were directed to visit an HF for PQ

Variable	Value
Gender of patients—n (%)	
Male	167 (54.0)
Female	142 (46.0)
Age of patients, years—median [IQR]	7 [4–10]
Age of caregivers, years—median [IQR]	34 [28–42]
Educational level of caregivers, years—median [IQR]	6 [6–8]
Marital status of caregivers—n (%)	
Married	286 (92.6)
Unmarried	6 (1.9)
Divorced or separated	10 (3.2)
Widowed	7 (2.3)
Household size—median [IQR]	6 [5–7]
Missing—n	1
Wealth status—n (%)	
Lower	107 (36.0)
Middle	90 (30.3)
Upper	100 (33.7)
Missing	12
Transportation cost to HFs, Kina^a^—median [IQR]	0 [0–4]
Missing—n	4
Place of initial visit—n (%)	
VHV	168 (54.4)
HF	141 (45.6)

Shapiro–Wilk’s normality test found all continuous variables to be non-normally distributed

*VHV* village health volunteer, *HF* formal health facility, *IQR* interquartile range

^a^PNG Kina 1 = USD 0.37 in 2015

### Determinants of PQ prescription during second visit to HF

Using a three-level multilevel random intercept logistic regression model, place of initial visit and transportation cost to HFs were factors significantly associated with obtaining a PQ among children with potential non-falciparum malaria. Patients who visited VHVs at initial visit were less likely to be prescribed PQ during the second visits to HFs (OR = 1.92, 95% CI 1.00–3.69, *p* = 0.049), and higher transportation cost to HFs was associated with lower odds of PQ prescription (OR = 0.85, 95% CI 0.76–0.94, *p* = 0.002) (Table 2).

**Table 2:** Three-level logistic regression of factors associated with PQ prescription during the second visit to HF

Variable	OR (95% CI)	p-value
Gender of patients (female = 0/male = 1)	1.23 [0.68, 2.23]	0.49
Age of patients (year)	1.05 [0.96, 1.15]	0.31
Age of caregivers (year)	1.03 [0.99, 1.06]	0.16
Educational level of caregivers (year)	1.02 [0.92, 1.14]	0.66
Marital status of caregivers (married = 1)		
Unmarried	1.08 [0.17, 6.88]	0.94
Divorced/separated	1.78 [0.37, 8.52]	0.47
Widowed	0.25 [0.03, 2.19]	0.21
Household size	0.92 [0.81, 1.04]	0.19
Wealth status (Lower = 1)		
Middle	0.82 [0.40, 1.68]	0.59
Upper	0.73 [0.35, 1.52]	0.41
Transportation cost to HF (PNG Kina)	0.85 [0.77, 0.94]	0.002
Place of initial visit (VHV = 0/HF = 1)	1.94 [1.01, 3.71]	0.047

Transportation cost to the HF (*p* = 0.002) and place of initial visit (*p* = 0.047) were significantly affected PQ prescription during the second visit to an HF. The significance level was *p* < 0.05

*VHV* village health volunteer, *HF* health facility, *OR* odds ratio, *CI* confidence interval

## Discussion

The study’s main findings were as follows. First, the overall proportion of patients with potential non-falciparum malaria who received PQ was 50%. Second, the proportion of patients who received PQ during the second visits to HFs was 44%. Third, a multilevel logistic analysis suggested that the initial visit to a VHV and higher transportation costs were inversely associated with the PQ prescription during the second visit to an HF.

The 50% proportion of patients who received PQ was lower than that found by previous studies in the Peruvian Amazon (78%) [22] and in Indonesia (65%) [23]. However, these studies have investigated proportions of patients who received PQ treatment from medical records or patients’ self-reports in medical facilities where patients visited. Thus, cases of referral patients are not included, and this may have resulted in a higher proportion of patients who reached PQ treatment.

The present study found children diagnosed with potential non-falciparum malaria in HFs were more likely to obtain PQ during their second HF visit than those diagnosed by VHVs (Table 2). These results may have been due to VHV’s inadequacy of specific instructions to patients. In PQ treatment-adherence studies in the Brazilian Amazon, patients who received adequate prescription instruction by health-care providers were more likely to adhere to treatment [38]. In the study area, VHVs did not receive refresher training after they completed initial short-term training for malaria case management. In addition, VHVs were not authorized to prescribe PQ, whereas HF health-care providers prescribe PQ as part of their daily work, so VHVs may not properly explain to the patients about the importance of the visit an HF for PQ. Moreover, disease severity may affect HF visits for PQ. Several studies showed that treatment seeking in the formal health sector was associated with disease severity [39, 40], and from that perspective, more severe patients may consult with HFs than VHVs and are likely to continue PQ treatment at HFs.

In the present study, higher transportation costs were negatively associated with PQ prescription during the second visit to an HF (Table 2). Transportation costs may substantially impact the study population and pressure people to sacrifice other living expenses. Although no studies have evaluated the effect of transportation costs on access to PQ treatment, several have shown association between transportation cost and appropriate treatment-seeking behaviour in an acute phase [41, 42]. In addition, this aligns with results from previous studies focusing on retention rates and transportation costs among patients with human immunodeficiency virus (HIV) in rural Haiti and Uganda [43, 44].

To improve access to HFs for PQ, structural and economic barriers may be critical elements to consider. Thus, four possible policy options are proposed: (1) decentralizing medical care by enabling VHVs to prescribe PQ, (2) free public transportation to HFs, (3) PQ provision through routine outreach activities, and (4) concurrent prescription of AL and PQ.

First, decentralization of PQ treatment could be a promising solution. In rural PNG, VHVs have been identified as a key basic health-care provider and have diagnosed and treated acute malaria patients. VHVs are the most accessible of health-care personnel for residents; therefore, empowering them to prescribe PQ could be an efficient and feasible way to improve access. For VHVs, a decentralized treatment of PQ would give greater responsibility in treating patients with vivax malaria. Among residents, this practice could increase interest about and understanding of PQ. Additionally, VHVs that are closer to residents have potential to more effectively improve a therapeutic course via directly observed therapy, such as medication adherence and monitoring risk of haemolysis among people with G6PD deficiency. However, in terms of G6PD deficiency, as haemolytic risk is considered to be high in PNG [45, 46], sufficient preliminary assessment of tolerability and safety of PQ administration must first be conducted. In cases where nonmedical workers intervene in PQ treatment, screening using point-of-care G6PD testing is recommended for the risk management of haemolysis in low-resource settings [47].

Second, free transportation could be a helpful incentive to revisit HFs. Currently, in PNG, examinations and PQ treatment are free at HFs, so the only direct cost for patients and their families is transportation. In a study conducted in Tanzania, funding assistance to cover the transportation cost of returning to the hospital was associated with improved adherence to follow-up protocols among children diagnosed with tuberculosis [48]. Similarly in Malawi, transportation cost to a hospital was associated with improved long-term follow-up in antiretroviral treatment among adult HIV patients co-infected with tuberculosis [49]. However, cost-effectiveness assessment, including management cost, would be necessary for the perspective of sustainability in PNG.

Third, the availability of routine outreach activities could improve PQ treatment for rural residents. In the study area, childcare-outreach services visit each village every 2 months to administer vaccinations and health examinations for children, providing PQ treatment as part of the outreach services may therefore be partly effective for reducing vivax transmission. PNG has a short-term relapsing strain in which multiple relapses are very common (similar to the Chesson strain; with which 80% of relapses occur within 1 month after the treated primary infection) [50]. Even if PQ treatment is given more than 2 months after the disease onset by the outreach services, it is still possible to reduce the number of relapses and infection transmission. Furthermore, during routine visits to each village, health-care professionals can monitor the signs and symptoms of possible adverse reactions for patients already given PQ treatment, and can also mentor the VHVs and share with them relevant health-care information on PQ-induced adverse reactions. These policies may lead to better implementation of treatment for needy children infected with vivax malaria.

Last, concurrent prescription of AL and PQ, which has been adopted in Vanuatu could increase the proportion of patients obtaining PQ treatment: PQ is prescribed with AL once and started on the same day with AL. Vanuatu’s national guidelines recommended the routine testing of G6PD deficiency before PQ administration, and require clinical monitoring at higher level health facilities for patients with an unknown G6PD status at the time they received PQ treatment [51]. However, concurrent prescription needs some consideration. Eventual adherence, including 14 days oral administration, is unclear unless differences are compared between patients prescribed PQ at the initial visit and at the second visit. Moreover, little is known about the safety and efficacy of concurrent use of AL and PQ. Some partner blood schizonticides, including AL may affect PQ metabolism via inhibition of cytochrome P450-2D6 activity [1, 52]. For these reasons, health facilities should establish emergency responses to G6PD deficiency, such as blood transfusion for an acute haemolytic event. Furthermore, patients should be given instructions about the signs and symptoms of possible adverse reactions (such as dark-coloured urine, gastrointestinal symptoms and fatigue), emphasizing the necessity of a return visit if the patient experiences any of those adverse reactions.

Apart from the possible changes in policy as mentioned above, current PQ administration in the study setting may be problematic; it was found that PQ was prescribed for patients with an RDT result of “falciparum malaria or falciparum malaria with infections of other malaria species”, although the national guidelines recommend that PQ should be prescribed only when confirmed as vivax and/or ovale malaria [34]. Therefore, the observed PQ treatment may lead to overuse of PQ and any subsequent unnecessary adverse reactions. It is rather preferable to establish a referral system to ensure a definitive diagnosis using a microscope or *P. vivax*-specific bivalent RDT toward the elimination stage of vivax malaria. In the present study setting, it is feasible to make a blood film diagnosis for malaria at the HF and refer to the provincial hospital for microscopic examination. In this situation, it is more important to strengthen a referral system between VHVs and HFs for elimination stage of vivax malaria at a community level.

The present study has some limitations. First, PQ prescription during the second visits to HFs among children with potential non-falciparum malaria was assessed through inspection of child health-record books. Possibly, health-care providers did not always enter patients’ information in record books, and therefore, the proportion of children completing the requisite HF visits for PQ could be biased. However, this study is perceived to be more credible than a previous study evaluating the proportion of PQ prescriptions through patients’ self-reports [23]. Second, access to PQ treatment was defined as obtaining PQ during a second HF visit following diagnosis of potential non-falciparum malaria. The word “access” originally has three dimensions, including availability, affordability, and acceptability [53]. However, the present study mainly focused on the former two aspects, excluding acceptability—such as perception of malaria disease and its severity, belief in the effectiveness of medicine, knowledge and perception of the risk of its adverse reactions, characteristics of health providers, and physician–patient relationships—that could affect the second visit to HF and subsequent PQ prescription. Third, indirect costs (the total costs of travel, waiting, and consultation time) could not be included in the analysis because it was not possible to obtain sufficient information to estimate these costs. The effect of costs on the PQ access may be overestimated. Fourth, the descriptive statistics of several explanatory variables showed significant differences between the excluded and the remaining groups, although there was no significant difference in the median of the significant determinant of PQ access (i.e., the transportation cost to the HFs). This could have resulted in a selection bias on the results of the logistic regression analysis. Fifth, because not all communities in rural area of PNG have working VHV programmes, generalizing these results to the rest of the region might be difficult. However, this study might provide useful information for policy planning in regions that face similar problems.

Despite these limitations, the present study showed that there is inadequate access to PQ treatment in rural areas of PNG. Given the continuing global effort toward eliminating vivax malaria, further studies exploring PQ treatment from the stand point of community case management is important where VHVs are often the first contact point, which could contribute to establishment of better strategies for vivax malaria elimination in the Asia and Pacific region.

## Conclusions

Few children obtained PQ treatment during their second visits to HFs following diagnosis of potential non-falciparum malaria in rural PNG. These findings suggest that inadequate referrals from VHVs to HFs, and economic barriers of traveling to HFs, were critical factors in the access to PQ treatment. Vivax malaria-control policy making should focus on ways to increase access to PQ, such as the decentralization of treatment, free transportation, enhancement of outreach activity, and concurrent prescription of AL and PQ.

## Additional file

**Additional file 1: Table S1.** Comparison of descriptive statistics between the remaining and the excluded groups.

## Abbreviations

AL: artemether–lumefantrine
HF: formal health facility
HIV: human immunodeficiency virus
MHC: mobile health clinic
PCA: principal component analysis
PNG: Papua New Guinea
PQ: primaquine
RDT: rapid diagnostic test
VHV: village health volunteer
CI: confidence interval
WHO: World Health Organization
