# Supplementary material for: Factors associated with children’s health facility visits for primaquine treatment in rural Papua New Guinea
Source: Malar J. 2019 Feb 20;18:42. doi: 10.1186/s12936-019-2675-4 (PMC6383275; doi:10.1186/s12936-019-2675-4)
Supplement: Supplementary file 1 — Additional file 1: Table S1. Comparison of descriptive statistics between the remaining and the excluded groups. [file 12936_2019_2675_MOESM1_ESM.docx]

**Table S1:**

Comparison of descriptive statistics between the remaining and the excluded groups

|  | **Remaining group** | **Excluded group** |  |
| --- | --- | --- | --- |
| **Variable** | **(n=1932)** | **(n=835)** | **P value** |
| Gender of patients – n (%)  Male  Female | 1027 (53) 905 (47) | 419 (50) 416 (50) | 0.15 |
| Age of patients, years – median [IQR]  Missing – n | 6 [3-10]  1 | 8 [4-11]  3 | <0.01 |
| Age of caregivers, years – median [IQR] | 33 [28-40] | 35 [29-41] | 0.018 |
| Educational level of caregivers, years  – median [IQR]  Missing – n | 6 [6-8]  7 | 6 [6-8]  5 | 0.47 |
| Marital status of caregivers – n (%)  Married  Unmarried  Divorced or separated  Widowed  Missing | 1787 (92.6)  38 (2.0)  59 (3.1)  45 (2.3)  3 | 755 (90.8)  15 (1.8)  24 (2.9)  38 (4.6)  3 | 0.018 |
| Household size – median [IQR]  Missing – n | 6 [5-8]  6 | 7 [5-8]  6 | <0.01 |
| Wealth status – n (%)  Lower  Middle  Upper  Missing – n | 718 (38.8) 552 (29.7) 591 (31.8)  71 | 300 (37.9) 202 (25.5) 289 (36.5)  44 | 0.027 |
| Transportation cost to HFs, Kina*  – median [IQR]  Missing – n | 0 [0-10] 37 | 0 [0-10] 46 | 0.39 |
| Place of initial visit – n (%) | N/A | N/A | N/A |

Shapiro-Wilk’s normality test found all continuous variables to be non-normally distributed.

Wilcoxon rank-sum tests were used to compare medians of continuous variables and chi-square tests were applied to compare proportions of categorical variables. The significance level was *p* < 0.05.

* PNG Kina 1 = USD 0.37 in 2015

Abbreviations: HFs, health facility; OR, odds ratio; CI, confidence interval; IQR, interquartile range; N/A, not available
